# Covert Communications via Full-Duplex User Relaying

**DOI:** 10.3390/s25123614

**Published:** 2025-06-09

**Authors:** Jong Yeol Ryu, Jung Hoon Lee

**Affiliations:** 1 Department of AI Information Engineering, Gyeongsang National University, Jinju 52828, Republic of Korea; jongyeol_ryu@gnu.ac.kr; 2 Department of Electronics Engineering and Applied Communications Research Center, Hankuk University of Foreign Studies (HUFS), Yongin 17035, Republic of Korea

**Keywords:** covert communication, user relaying, signal cancellation, power allocation, covert rate

## Abstract

In this paper, we investigate a covert communication system with a full-duplex decode-and-forward (DF) relay and introduce a user-relaying scheme that maximizes the covert rate while ensuring the covertness requirement. In our system model, Alice (transmitter) sends regular data to Carol (regular user) and occasionally embeds covert data for Bob (covert user). Meanwhile, Willie (warden) monitors for covert transmissions. Carol assists Alice by acting as a full-duplex DF relay, decoding both data types via successive interference cancellation and relaying covert data using phase steering and power allocation to confuse Willie. Our proposed scheme adopts a novel approach in which the covert data received by Willie is perfectly canceled, optimizing Alice’s and Carol’s transmissions to maximize the covert rate while keeping Willie’s detection probability below a given threshold.

## 1. Introduction

As wireless security becomes increasingly critical, covert communication has emerged as a key technique for securing future wireless systems [1]. While conventional physical-layer security techniques aim to protect messages from eavesdroppers [2], covert communication techniques focus on preventing exposure of the transmission’s presence to wardens. Consequently, covert communications can offer stronger security than conventional physical-layer security techniques. Recently, due to their potential for various applications, such as military communications, covert communication techniques have been intensively investigated [3,4,5,6,7,8,9,10].

The information-theoretic limit of covert communication was studied in [3]. The authors derived the square root law for additive white Gaussian noise (AWGN) channels, showing that the transmitter can covertly transmit $O(\sqrt{n})$ bits over *n* channel uses. Various conditions for achieving a positive covert rate were discussed in [4,5]; it was demonstrated that a positive covert rate can be obtained when the warden has uncertainty about the channel state information [4] or the background noise power [5]. Friendly jammer-assisted covert communication systems were considered in [6,7]. The authors of [6] optimized the covert rate in scenarios where wardens and jammers are randomly distributed across the network. Also, the authors of [7] introduced a threshold-based uncoordinated jammer selection scheme to counteract the warden’s efforts.

To further increase the covert rate, covert communication systems leveraging relay nodes were investigated in [8,9,10]. In these studies, the relay plays a dual role, that is, as a helper, cooperatively forwarding covert data to enhance the quality of the covert signal, and as a friendly jammer. The authors of [8] proposed full-duplex amplify-and-forward (AF) relaying for covert rate maximization. In [8], the relay node acts as a jammer when the transmitter does not send covert data and as an AF relay when covert data is transmitted. The authors optimized the relay power to maximize the covert rate while satisfying covertness constraints. A decode-and-forward (DF) relay-assisted covert communication system was considered in [9]. The authors jointly optimized the beamformers at the transmitter and the relay to exploit the benefits of both the direct and relay links. Recently, covert communication systems coexisting with relays and jammers were introduced in [10]. In [10], relay selection schemes and corresponding cooperative jamming strategies were proposed, and power control was optimized for covert rate maximization. The authors of [11] showed that the covert transmission of O(n) bits over *n* channel uses can be achieved with the help of a relay under channel uncertainty. Also, the authors of [12] optimized covert communications with multiple relays in the presence of an active warden.

In this paper, we propose a novel approach to covert communication using a user relay, where the relay’s role is to entirely cancel the covert signal at the warden through joint power optimization with the transmitter. In previous literature [8,9,10,11,12], the role of the relay in covert communications has been somewhat limited. In most works, the relay either transmits jamming signals to confuse Willie’s detection or functions as a conventional relay by forwarding Bob’s signal to enhance his received signal strength. In contrast, our proposed approach designs the relay to perfectly nullify the covert signals at Willie. This cancellation enables Alice to allocate more power to the covert data, thereby increasing the covert rate while still satisfying the covertness constraint. To the best of our knowledge, this represents a novel approach to utilizing a relay in covert communication, which has not been explored in existing studies. We first formulate a joint optimization problem for the transmitter and the user relay, incorporating constraints on the user relay’s quality of service (QoS), covert signal cancellation at the warden, and the covertness requirement. We then derive closed-form solutions for the optimal power allocation of the transmitter and the user-relaying strategy that maximizes the covert data rate. The numerical results demonstrate that our proposed approach significantly enhances the covert rate compared to other reference schemes.

## 2. System Model

### 2.1. System Configuration

**Figure 1:**
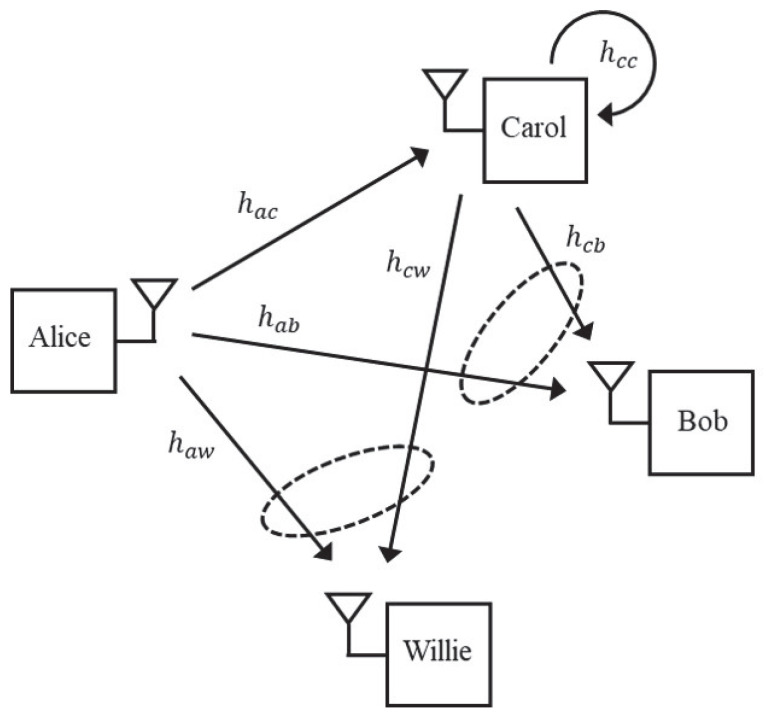
Covert communication with user relaying.

Our system model is illustrated in Figure 1. We consider a covert communication system, which comprises Alice (a transmitter), Bob (a covert user), Carol (a regular user, and Willie (a warden). Alice consistently transmits regular data to Carol, while occasionally transmitting covert data to Bob by superimposing it onto the regular data. At the same time, Willie observes the received signal and attempts to identify the covert data transmission between Alice and Bob. In our system model, Carol is a full-duplex DF relay [13,14,15] and assists Alice’s covert data transmission to Bob. During covert data transmission, Carol first decodes her own data and then decodes the superimposed covert data using successive interference cancellation (SIC). Since Carol is a regular user who is always served by Alice, regardless of whether Alice is transmitting covert data, we assume that Carol first decodes her desired signal rather than the covert data. From the perspective of power-domain non-orthogonal multiple access (NOMA), it is natural that the signal with the higher power in the superposed signal is decoded first. In our case, it is more likely that Carol’s received power for regular data is larger than the received power for covert data. Therefore, it makes sense for Carol to decode her desired data first. Then, Carol transmits the covert data to Bob after applying appropriate phase steering and power allocation.

We assume that every node is equipped with a single antenna, and denote by {$h_{ab}$, $h_{ac}$, and $h_{aw}$} the channels from Alice to {Bob, Carol, and Willie}, respectively. Also, we denote by {$h_{cb}$ and $h_{cw}$} the channels from Carol to {Bob and Willie}, respectively. The self-interference channel of Carol caused by full-duplex relaying is denoted by $h_{cc}$. Each channel is modeled by a Rayleigh fading channel, such that $h_{ij}∼\mathrm{CN}\left(0,\gamma_{ij}\right)$ for i,j∈{a,b,c,w}, where $\gamma_{ij}$ is the average channel gain of $h_{ij}$, i.e., $\gamma_{ij}≜E{\left|h_{ij}\right|}^{2}$.

### 2.2. Signal Models

In our system model, Alice may transmit either only the regular message (Carol’s) or both the regular message and the covert message to Bob (Bob’s). These correspond to two hypotheses, $H_{0}$ (only the regular message) and $H_{1}\left(≜H_{0}^{c}\right)$ (regular and covert messages). Thus, the transmit signal at Alice can be represented by

(1) $$\begin{array}{cc} \; & H_{0}:\;x=\sqrt{P_{a}}x_{c}\;\;\;\;\;\;\;\;\;\;\;\;\;\;\;\;\;\;\;\;\;\;\;\;\;\;\;\;\;\;\;\;\; \end{array}$$

(2) $$\begin{array}{cc} \; & H_{1}:\;x=\sqrt{\left(1-\alpha \right)P_{a}}x_{c}+\sqrt{\alpha P_{a}}x_{b}, \end{array}$$

where $x_{c}\in C$ and $x_{b}\in C$ denote the regular data for Carol and the covert data for Bob, respectively, such that $E|x_{c}{|}^{2}=E{\left|x_{b}\right|}^{2}=1$. Also, $P_{a}$ denotes Alice’s transmit power budget, and α∈[0,1] denotes the power portion for the covert data.

As a result, the received signal at Carol becomes

(3) $$\begin{array}{cc} \; & H_{0}:\;y_{c}=\sqrt{P_{a}}h_{ac}x_{c}+n_{c}\;\;\;\;\;\;\;\;\;\;\;\;\;\;\;\;\;\;\;\;\;\;\;\;\;\;\;\;\;\;\;\;\;\;\;\;\;\;\;\;\;\;\;\;\; \end{array}$$

(4) $$\begin{array}{cc} \; & H_{1}:\;y_{c}=h_{ac}\left(\sqrt{\left(1-\alpha \right)P_{a}}x_{c}+\sqrt{\alpha P_{a}}x_{b}\right)+v_{c}+n_{c}, \end{array}$$

where $v_{c}$ denotes the self-interference caused by full-duplex relaying and $n_{c}∼\mathrm{CN}\left(0,\sigma_{c}^{2}\right)$ represents circularly symmetric complex Gaussian noise at Carol. When $H_{0}$ holds, Carol’s achievable rate becomes

(5) $$\begin{array}{c} H_{0}:\;R_{c}=\log_{2}\left(1+\frac{P_{a}{\left|h_{ac}\right|}^{2}}{\sigma_{c}^{2}}\right). \end{array}$$

On the other hand, when $H_{1}$ holds, Carol first decodes her desired signal $x_{c}$ while treating $x_{b}$ as interference, resulting in the achievable rate given by

(6) $$\begin{array}{c} H_{1}:\;R_{c}=\log_{2}\left(1+\frac{\left(1-\alpha \right)P_{a}{\left|h_{ac}\right|}^{2}}{\alpha P_{a}|h_{ac}{|}^{2}+p_{c}{\left|h_{cc}\right|}^{2}+\sigma_{c}^{2}}\right), \end{array}$$

where Carol uses a transmit power $p_{c}$ for user relaying, and, thus, the self-interference power is given by $p_{c}{\left|h_{cc}\right|}^{2}$. Note that, since perfect self-interference cancellation is difficult to achieve, the residual self-interference power is modeled by the self-interference channel gain $|h_{cc}{|}^{2}$, where $|h_{cc}{|}^{2}=0$ corresponds to perfect self-interference cancellation [16]. In this case, Carol’s QoS should be guaranteed during the covert data transmission as follows (assuming that $P_{a}$ is large enough so that Carol’s QoS is achieved when $H_{0}$ holds).

(7) $$\begin{array}{c} R_{c}\geq R\;\mathrm{for}H_{1}, \end{array}$$

where *R* denotes the minimum required rate for Carol. Then, by applying SIC, Carol’s achievable rate for covert data becomes

(8) $$\begin{array}{c} H_{1}:\;R_{b}^{\mathrm{Carol}}=\log_{2}\left(1+\frac{\alpha P_{a}{\left|h_{ac}\right|}^{2}}{p_{c}{\left|h_{cc}\right|}^{2}+\sigma_{c}^{2}}\right). \end{array}$$

In our scheme, Carol assists with the covert transmission to Bob by acting as a DF user relay; Carol transmits $x_{b}$ to Bob using the transmit power, $p_{c}$, after applying a phase steering parameter, $w_{c}$, i.e., $\sqrt{p_{c}}w_{c}x_{b}$. In this case, $p_{c}\leq P_{c}$ and $|w_{c}|=1$ must be satisfied, where $P_{c}$ denotes Carol’s maximum transmit power budget.

Therefore, the received signal at Bob becomes as follows:

(9) $$\begin{array}{cc} H_{0}:\;y_{b} & =\sqrt{P_{a}}h_{ab}x_{c}+n_{b}\;\;\;\;\;\;\;\;\;\;\;\;\;\;\;\;\;\;\;\;\;\;\;\;\;\;\;\;\;\;\;\;\;\;\;\;\;\;\;\;\;\;\;\;\;\;\;\;\;\;\;\;\;\;\;\;\;\;\;\; \end{array}$$

(10) $$\begin{array}{cc} H_{1}:\;y_{b} & =h_{ab}\left(\sqrt{\left(1-\alpha \right)P_{a}}x_{c}+\sqrt{\alpha P_{a}}x_{b}\right)+\sqrt{p_{c}}h_{cb}w_{c}x_{b}+n_{b}\\ \; & =h_{ab}\sqrt{\left(1-\alpha \right)P_{a}}x_{c}+{\tilde{h}}_{b}x_{b}+n_{b}, \end{array}$$

where $n_{b}∼\mathrm{CN}\left(0,\sigma_{b}^{2}\right)$ represents complex Gaussian noise at Bob, and ${\tilde{h}}_{b}$ denotes the effective channel at Bob for the covert data given by

$${\tilde{h}}_{b}≜\sqrt{\alpha P_{a}}h_{ab}+\sqrt{p_{c}}h_{cb}w_{c}.$$

In the DF relaying system, since the data should be decoded by both the relay and the destination, the achievable rate is bounded by the minimum of the achievable rates at these two nodes [17]. Therefore, from (8) and (10), we obtain the achievable rate of Bob as follows:

(11) $$\begin{array}{c} R_{b}=\log_{2}\left(1+\min\left\{\frac{\alpha P_{a}{\left|h_{ac}\right|}^{2}}{p_{c}{\left|h_{cc}\right|}^{2}+\sigma_{c}^{2}},\frac{|{\tilde{h}}_{b}{|}^{2}}{\left(1-\alpha \right)P_{a}{\left|h_{ab}\right|}^{2}+\sigma_{b}^{2}}\right\}\right). \end{array}$$

Also, the received signal at Willie is given by

(12) $$\begin{array}{cc} H_{0}:\;y_{w} & =\sqrt{P_{a}}h_{aw}x_{c}+n_{w}\;\;\;\;\;\;\;\;\;\;\;\;\;\;\;\;\;\;\;\;\;\;\;\;\;\;\;\;\;\;\;\;\;\;\;\;\;\;\;\;\;\;\;\;\;\;\;\;\;\;\;\;\;\;\;\;\;\;\;\; \end{array}$$

(13) $$\begin{array}{cc} H_{1}:\;y_{w} & =h_{aw}\left(\sqrt{\left(1-\alpha \right)P_{a}}x_{c}+\sqrt{\alpha P_{a}}x_{b}\right)+\sqrt{p_{c}}h_{cw}w_{c}x_{b}+n_{w}\\ \; & =\sqrt{\left(1-\alpha \right)P_{a}}h_{aw}x_{c}+{\tilde{h}}_{w}x_{b}+n_{w}, \end{array}$$

where $n_{w}∼\mathrm{CN}\left(0,\sigma_{w}^{2}\right)$ is the complex Gaussian noise at Willie, and ${\tilde{h}}_{w}$ is an effective channel experienced by the covert data $x_{b}$ at Willie, given by

(14) $$\begin{array}{c} {\tilde{h}}_{w}≜\sqrt{\alpha P_{a}}h_{aw}+\sqrt{p_{c}}h_{cw}w_{c}. \end{array}$$

### 2.3. Covert Communication Requirement

According to the Neyman–Pearson criterion, Willie decides between the hypotheses based on the received signal power measured by a radiometer (or power detector) as

(15) $$\begin{array}{c} Y_{w}={\left|y_{w}\right|}^{2}\underset{D_{0}}{\overset{D_{1}}{≷}}\phi , \end{array}$$

where $D_{0}$ and $D_{1}$ denote Willie’s decisions to accept hypotheses $H_{0}$ and $H_{1}$, respectively, and ϕ is a decision threshold.

In general, the priori probabilities of $H_{0}$ and $H_{1}$ are assumed to be equal [9]. In this case, the detection error probability at Willie becomes

(16) $$\begin{array}{cc} \xi & ≜\mathrm{Pr}\left(D_{1}|H_{0}\right)+\mathrm{Pr}\left(D_{0}|H_{1}\right), \end{array}$$

where $\mathrm{Pr}\left(D_{1}|H_{0}\right)$ and $\mathrm{Pr}\left(D_{0}|H_{1}\right)$ correspond to the false alarm and the miss detection probabilities, respectively.

In this paper, we consider that covert communication is achieved when the detection probability of Willie is less than a predefined value ϵ, which we refer to as a covertness requirement. In other words, to achieve covert communication, the detection error probability of Willie should be larger than or equal to 1−ϵ, i.e., ξ≥1−ϵ.

Let $P_{0}$ and $P_{1}$ denote the probability distributions of Willie’s observation (i.e., $y_{w}$) under $H_{0}$ and $H_{1}$, respectively. Then, by taking the optimal value of ϕ, which minimizes the detection error probability, Willie’s corresponding detection error probability in (16) can be represented by the following [3]:

(17) $$\begin{array}{cc} \xi & =1-V\left(P_{0},P_{1}\right), \end{array}$$

where $V\left(P_{0},P_{1}\right)$ denotes the total variation between $P_{0}$ and $P_{1}$. However, in general, it is difficult to calculate $V\left(P_{0},P_{1}\right)$ analytically. Thus, using Pinsker’s inequality [18], we obtain a lower bound for the detection error probability given by

(18) $$\begin{array}{cc} \; & \xi \geq 1-\sqrt{\frac{1}{2}D\left(P_{0}∥P_{1}\right)}, \end{array}$$

In (18), $D\left(P_{0}∥P_{1}\right)$ denotes the Kullback–Leibler (KL) divergence from $P_{0}$ to $P_{1}$ given by

(19) $$D\left(P_{0}∥P_{1}\right)≜\int_{-\infty }^{\infty }p_{0}\left(y_{w}\right)\ln\frac{p_{0}\left(y_{w}\right)}{p_{1}\left(y_{w}\right)}dy,$$

where $p_{0}\left(y_{w}\right)$ and $p_{1}\left(y_{w}\right)$ are the probability densities of $P_{0}$ and $P_{1}$, respectively.

From (12) and (13), the probability densities $p_{0}\left(y_{w}\right)$ and $p_{1}\left(y_{w}\right)$ can be obtained as

(20) $$\begin{array}{cc} p_{0}\left(y_{w}\right) & =\frac{1}{\pi (P_{a}|h_{aw}{|}^{2}+\sigma_{w}^{2})}\exp\left(-\frac{{\left|y_{w}\right|}^{2}}{P_{a}{\left|h_{aw}\right|}^{2}+\sigma_{w}^{2}}\right)\;\;\; \end{array}$$

(21) $$\begin{array}{cc} p_{1}\left(y_{w}\right) & =\frac{1}{\pi (\left(1-\alpha \right)P_{a}|h_{aw}{|}^{2}+|{\tilde{h}}_{w}{|}^{2}+\sigma_{w}^{2})}\\ \; & \;\times \exp\left(-\frac{{\left|y_{w}\right|}^{2}}{\left(1-\alpha \right)P_{a}|h_{aw}{|}^{2}+{\left|{\tilde{h}}_{w}\right|}^{2}+\sigma_{w}^{2}}\right). \end{array}$$

Substituting (20) and (21) into (19), the KL divergence can be summarized as follows:

(22) $$\begin{array}{cc} D(P_{0}∥P_{1}) & =\ln\left(\frac{\left(1-\alpha \right)P_{a}|h_{aw}{|}^{2}+{\left|{\tilde{h}}_{w}\right|}^{2}+\sigma_{w}^{2}}{P_{a}{\left|h_{aw}\right|}^{2}+\sigma_{w}^{2}}\right)\\ \; & \;+\frac{P_{a}{\left|h_{aw}\right|}^{2}+\sigma_{w}^{2}}{\left(1-\alpha \right)P_{a}|h_{aw}{|}^{2}+{\left|{\tilde{h}}_{w}\right|}^{2}+\sigma_{w}^{2}}-1. \end{array}$$

Also, from (18), the covertness constraint can be rewritten as

(23) $$\begin{array}{c} D\left(P_{0}∥P_{1}\right)\leq 2\epsilon^{2}. \end{array}$$

## 3. Problem Formulation and Our Proposed Solution

In this paper, our goal is to maximize Bob’s covert rate while satisfying both the covertness constraint given in (23) and the QoS constraint of Carol given in (7). Thus, we jointly optimize Alice’s transmission strategy (power allocation for covert data) and Carol’s relaying strategy (power allocation and phase steering) by solving the following problem.

(24) $$\begin{array}{cc} \left(P1\right)\underset{\alpha ,p_{c},w_{c}}{\mathrm{maximize}} & R_{b}\\ \;\;\;\;\;\;\;\;\;\;\;\;\;\;\;\;\;\;\mathrm{subject}\mathrm{to} & R_{c}\geq R, \end{array}$$

(25) $$\begin{array}{cc} \; & \;\;\;\;\;\;\;\;\;\;\;\;\;\;\;\;\;\;\;\;\;\;\;\;\;\;\;\;\;\;\;\;\;\;\;\;\;\;\;\;\;\;\;\;\;\;\;\;\;\;\;\;\;\;\;\;\;\;\;\;\;\;\;\;\;\;\;\;D\left(P_{0}∥P_{1}\right)\leq 2\epsilon^{2},\\ \; & \;\;\;\;\;\;\;\;\;\;\;\;\;\;\;\;\;\;\;\;\;\;\;\;\;\;\;\;\;\;\;\;\;\;\;\;\;\;\;\;\;\;\;\;\;\;\;\;\;\;\;\;\;\;\;\;\;\;\;\;\;\;\;\;\;\;\;\;0\leq \alpha \leq 1,p_{c}\leq P_{c},{\left|w_{c}\right|}^{2}=1. \end{array}$$

However, problem (P1) is hard to solve directly due to the complexity of the constraint (25). Thus, in our proposed scheme, Carol constructs the relaying signal such that the covert data is perfectly nullified at Willie’s end, resulting in ${\tilde{h}}_{w}=0$ in (13).

By adding the covert data cancellation constraint at Willie, we solve the alternative problem as follows:

(26) $$\begin{array}{cc} \left(P2\right)\underset{\alpha ,p_{c},w_{c}}{\mathrm{maximize}} & R_{b}\\ \mathrm{subject}\mathrm{to} & R_{c}\geq R,\\ & D\left(P_{0}∥P_{1}\right)\leq 2\epsilon^{2},\\ \; & 0\leq \alpha \leq 1,p_{c}\leq P_{c},{\left|w_{c}\right|}^{2}=1,\\ \; & {\tilde{h}}_{w}=0. \end{array}$$

To solve problem (P2), we first find Carol’s transmit power and phase steering, i.e., $p_{c}$ and $w_{c}$, from the constraint (26). Let the phase steering of Carol be $w_{c}≜e^{-j\theta_{\mathrm{steer}}}$. Then, we can rewrite ${\tilde{h}}_{w}$ in (14) as follows:

(27) $${\tilde{h}}_{w}=\sqrt{\alpha P_{a}}|h_{aw}|e^{j\theta_{aw}}+\sqrt{p_{c}}\left|h_{cw}\right|e^{j(\theta_{cw}-\theta_{\mathrm{steer}})},$$

where $\theta_{aw}$ and $\theta_{cw}$ are the phases of the channels $h_{aw}$ and $h_{cw}$, respectively. To satisfy (26), it should be satisfied in (27) that

$$\begin{array}{c} \sqrt{\alpha P_{a}}|h_{aw}|=\sqrt{p_{c}}\left|h_{cw}\right|\mathrm{and}\theta_{aw}=\theta_{cw}-\theta_{\mathrm{steer}}+\pi . \end{array}$$

Thus, we obtain Carol’s power allocation $p_{c}^{⋆}$ and phase steering $w_{c}^{⋆}≜e^{-j\theta_{\mathrm{steer}}^{⋆}}$ to perfectly nullify the covert signal received at Willie as follows:

(28) $$\begin{array}{cc} \; & p_{c}^{⋆}≜\alpha P_{a}\cdot \frac{|h_{cw}{|}^{2}}{|h_{aw}{|}^{2}}\\ \; & \theta_{\mathrm{steer}}^{⋆}≜\theta_{cw}-\theta_{aw}+\pi . \end{array}$$

Meanwhile, we can observe from (28) that Carol’s relaying power is coupled with α, which is the power portion for covert data at Alice.

Substituting $p_{c}^{⋆}$ and $w_{c}^{⋆}$ into (11), the covert rate $R_{b}$ becomes

(29) $$\begin{array}{c} R_{b}=\log_{2}\left(1+\min\left\{\frac{\alpha P_{a}{\left|h_{ac}\right|}^{2}}{\alpha P_{a}{\left|{\bar{h}}_{c}\right|}^{2}+\sigma_{c}^{2}},\frac{\alpha P_{a}{\left|{\bar{h}}_{b}\right|}^{2}}{\left(1-\alpha \right)P_{a}{\left|h_{ab}\right|}^{2}+\sigma_{b}^{2}}\right\}\right), \end{array}$$

where ${\bar{h}}_{c}≜\frac{|h_{cw}\left|\cdot \right|h_{cc}|}{|h_{aw}|}$ and ${\bar{h}}_{b}≜h_{ab}+\frac{|h_{aw}{|}^{2}}{|h_{cw}{|}^{2}}h_{cb}w_{c}^{⋆}$. In this case, based on the fact that $E|{\bar{h}}_{b}{|}^{2}\geq E{\left|h_{ab}\right|}^{2}$, with our proposed user-relaying scheme, we can observe that Bob enjoys a higher channel gain in the average sense.

Now, we determine $\alpha^{⋆}$, the optimal power portion for Alice’s covert data transmission. Since the covert rate given in (29) is an increasing function of α, the value of α should be chosen as large as possible, but it is restricted by three bounds.

First, in (28), Carol’s transmit power should satisfy $p_{c}^{⋆}\leq P_{c}$, so we have the first upper bound of α as

(30) $$\begin{array}{c} \alpha \leq {\bar{\alpha }}_{1}≜\frac{P_{c}}{P_{a}}.\frac{|h_{aw}{|}^{2}}{|h_{cw}{|}^{2}}. \end{array}$$

Second, from ${\tilde{h}}_{w}=0$, the KL divergence in (22) is simplified to

(31) $$D\left(P_{0}∥P_{1}\right)=\ln\left(\frac{\left(1-\alpha \right)P_{a}{\left|h_{aw}\right|}^{2}+\sigma_{w}^{2}}{P_{a}{\left|h_{aw}\right|}^{2}+\sigma_{w}^{2}}\right)+\frac{P_{a}{\left|h_{aw}\right|}^{2}+\sigma_{w}^{2}}{\left(1-\alpha \right)P_{a}{\left|h_{aw}\right|}^{2}+\sigma_{w}^{2}}-1.$$

The partial derivative of KL divergence in (31) with respect to α is satisfied, so that $\partial D(P_{0}∥P_{1})/\partial \alpha \geq 0$ for any α∈[0,1]. Thus, the KL divergence is a monotonically increasing function of α in the range of 0≤α≤1, and has the maximum value at α=1. Thus, the constraint $D\left(P_{0}∥P_{1}\right)\leq 2\epsilon^{2}$ yields the second upper bound of α as

(32) $$\begin{array}{c} \alpha \leq {\bar{\alpha }}_{2}, \end{array}$$

where ${\bar{\alpha }}_{2}=1$ if $D{\left(P_{0}∥P_{1}\right)}_{|\alpha =1}\leq 2\epsilon^{2}$, and

$${\bar{\alpha }}_{2}≜\left(\frac{P_{a}{\left|h_{aw}\right|}^{2}+\sigma_{w}^{2}}{P_{a}{\left|h_{aw}\right|}^{2}}\right)\left[1-\exp\left(W\left(-\frac{1}{e^{1+2\epsilon^{2}}}\right)+1+2\epsilon^{2}\right)\right],$$

otherwise, where W(x) is the Lambert *W* function.

Third, Carol’s QoS constraint $R_{c}\geq R$ yields the third upper bound of α as

(33) $$\begin{array}{c} \alpha \leq {\bar{\alpha }}_{3}≜\frac{P_{a}{\left|h_{ac}\right|}^{2}-\left(2^{R}-1\right)\sigma_{c}^{2}}{P_{a}\left(2^{R}|h_{ac}{|}^{2}+\left(2^{R}-1\right){\left|{\bar{h}}_{c}\right|}^{2}\right)}. \end{array}$$

As mentioned earlier, the covert rate given in (29) is an increasing function of α, so we should choose the value of α as large as possible. At the same time, the value of α is limited by three upper bounds, that is, (30), (32) and (33). Thus, we obtain the optimal value of α as follows:

(34) $$\begin{array}{c} \alpha^{⋆}=\min\left\{{\bar{\alpha }}_{1},{\bar{\alpha }}_{2},{\bar{\alpha }}_{3}\right\}. \end{array}$$

Note that our proposed scheme can be readily extended to the case when there are multiple user relays. When there are *K* user relays, we can consider the relay selection scheme based on our proposed scheme. Assuming that the user relay *k* is selected, we solve problem (P2) and obtain the optimal solution denoted by $\left(\alpha^{(k)⋆},p_{c}^{(k)⋆},w_{c}^{(k)⋆}\right)$. After solving problem (P2) for all *K* user relays, we choose the best user relay that maximizes the covert rate as follows:

(35) $$k^{⋆}≜\underset{k\in \{1,\ldots ,K\}}{\mathrm{argmax}}\;R_{b}^{\left(k\right)}\left(\alpha^{(k)⋆},p_{c}^{(k)⋆},w_{c}^{(k)⋆}\right),$$

where $R_{b}^{\left(k\right)}$ is the covert rate achieved by using the user relay *k*.

Note that although our system model may appear simple, the associated optimization problem remains complex. This is because the optimal covert transmission strategies of Alice and Carol must be jointly optimized and are not easily obtainable in closed form. Based on the key ideas presented in this work, we plan to extend the user-relaying concept to more generalized system models as part of our ongoing research.

## 4. Numerical Results

In this section, we evaluate our proposed user relaying with the phase steering scheme for covert communication. In our simulations, the average channel gain from Alice to Carol is set to $\gamma_{ac}=1$, and the average channel gains from Carol to Bob and Willie are set to $\gamma_{cb}=\gamma_{cw}=1$, unless otherwise specified. The average (direct) channel gains from Alice to Bob and Willie are set to $\gamma_{ab}=\gamma_{aw}=0.5$. The average channel gain of the self-interference is set to $\gamma_{cc}=0.2$ (we assume that the self-interference can be effectively reduced using efficient interference cancellation schemes, such as in [16]). All noise powers are set to be equal, with $\sigma_{b}^{2}=\sigma_{c}^{2}=\sigma_{w}^{2}=\sigma^{2}$. The transmit powers of Alice and Carol are assumed to be equal, so their transmit signal-to-noise ratio (SNR) becomes $\rho ≜\frac{P_{a}}{\sigma^{2}}=\frac{P_{c}}{\sigma^{2}}$. Also, we assume that the required rate of Carol is R=2 [bit/s/Hz]. For reference schemes, we consider (1) direct transmission without relaying and (2) full-duplex AF relaying for covert transmission [8].

**Figure 2:**
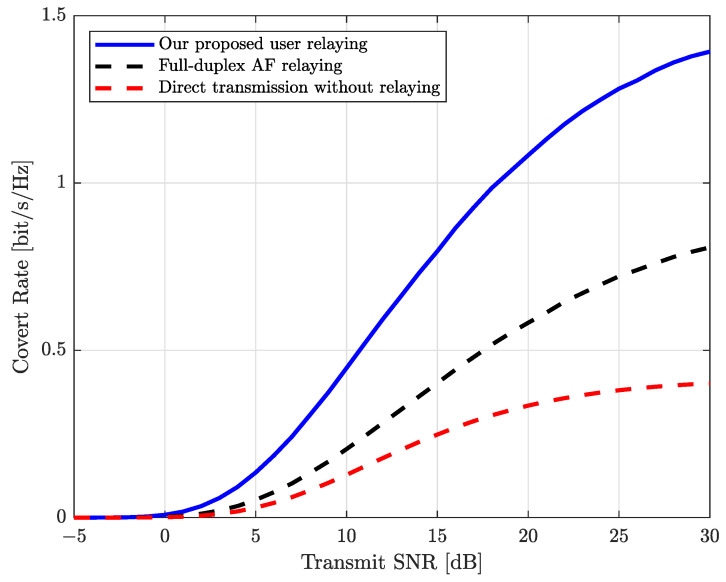
Covert rate vs. the transmit SNR (i.e., ρ).

In Figure 2, we first compare the covert rates for various schemes with respect to the transmit SNR, i.e., ρ. The covertness requirement is set to ϵ=0.2. In Figure 2, we can observe that the covert rate with every scheme increases as the transmit SNR increases, while our proposed scheme outperforms other reference schemes in all SNR regimes. In addition, we can observe that the performance enhancement of the proposed scheme becomes greater as the transmit SNR increases. This is because, as the transmit power of Alice and Carol increases, Carol can efficiently steer the phase and allocate power to completely cancel the covert signal at Willie. This allows Alice to allocate a larger portion of power for covert data transmission while satisfying the covertness requirement.

**Figure 3:**
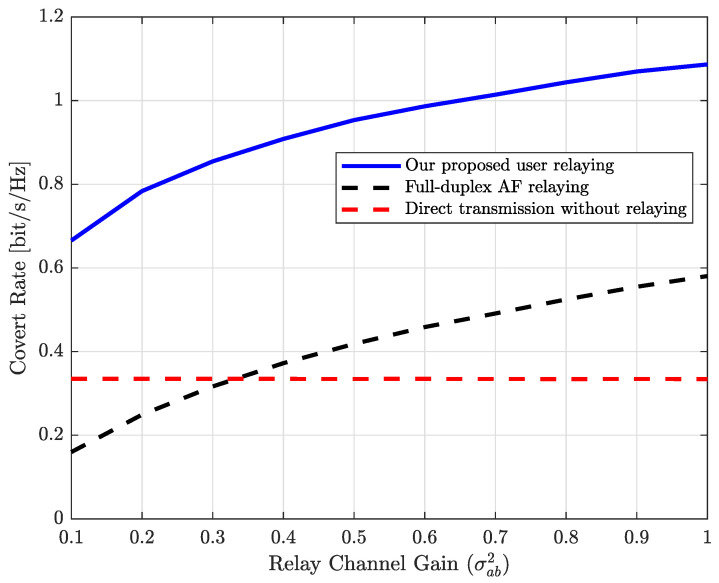
Covert rate vs. average relay channel gain (i.e., $\gamma_{cb}$).

Next, we show the impact of the average relay channel gain on the performance of various schemes. In Figure 3, the covert rates of various schemes are plotted with respect to the average relay channel gain, i.e., $\gamma_{cb}$. In this case, the transmit SNR is set to ρ=20 dB, while the covertness requirement is set to ϵ=0.2. From Figure 3, we can observe that, compared to direct transmission, the covert rate gain achieved through relaying increases with the average relay channel gain for both AF relaying and the proposed scheme. Meanwhile, AF relaying is inefficient when the relay channel strength is very weak (e.g., when $\gamma_{cb}<0.3$) compared to direct transmission without relaying. However, since we determine the power portion for the covert rate at Alice (i.e., α), the phase steering parameter, and the relaying power at Carol are jointly optimized for a given channel configuration, our proposed scheme outperforms direct transmission even when the relay channel is weak.

**Figure 4:**
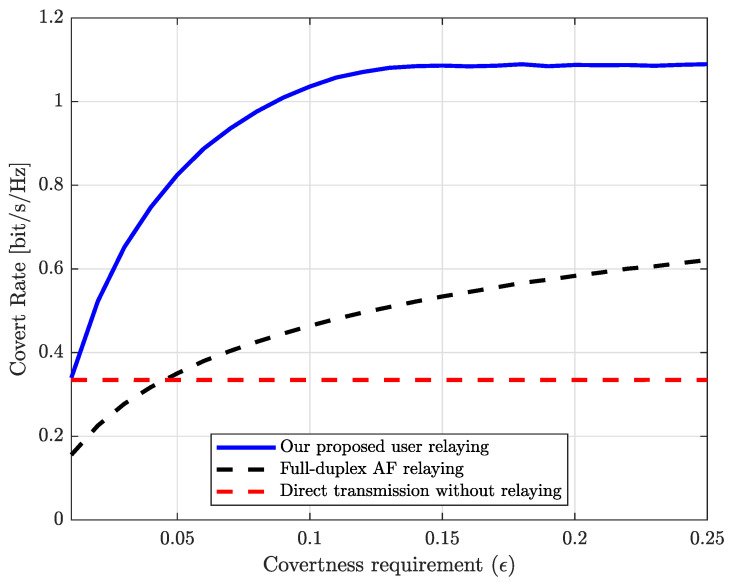
Covert rate vs. covertness requirement (i.e., ϵ).

We observe how the covertness requirement affects the performance of various schemes. In Figure 4, we show the covert rate of the proposed user relaying and the reference schemes with respect to the covertness requirement, i.e., ϵ. In this case, the transmit SNR is set to ρ=20 dB. For both schemes, we can observe that the covert rates increase as the covertness requirement increases, and our proposed scheme outperforms the AF relaying scheme for any covertness requirement. Since the power portion for the covert data (i.e., α) is bounded, as in (32), which is an increasing function with ϵ, Alice can allocate more power to the covert data as ϵ increases. Contrary to the covert rate of AF relaying, which slowly increases according to ϵ, for the region of ϵ>0.14, the power portion for the covert data is bounded by the QoS constraint of Carol in (33) rather than (32); hence, the covert rate of our proposed scheme is saturated.

**Figure 5:**
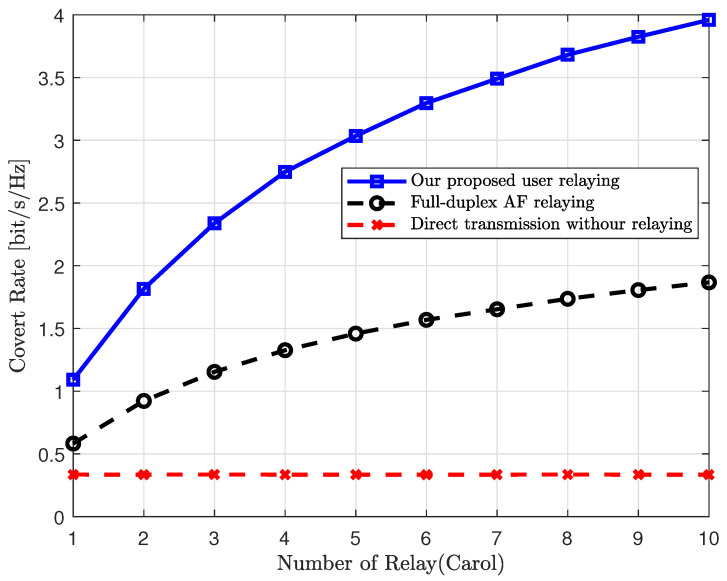
Covert rate vs. number of relays.

We also evaluate the performance improvement of our proposed scheme via relay selection when there are multiple user relays. In Figure 5, we present the covert rates of various schemes with respect to the number of user relays. In this figure, we set the transmit SNR and the covertness requirement as ρ=20 dB and ϵ=0.2, respectively. From Figure 5, we can observe that, unlike direct transmission, both our proposed relaying scheme and the AF relaying scheme achieve a higher covert rate as the number of user relays increases, benefiting from the diversity gain provided by multiple user relays. However, our proposed scheme achieves a significantly greater diversity gain compared to the AF relaying scheme. This is because the performance of our proposed scheme considers not only the channel gains but also the phase differences between the channels of Willie and the user relays.

## 5. Conclusions

In this paper, we propose a user-relaying scheme for covert communication networks, where a legitimate user, Carol, acts as a relay to assist with covert data transmission. Our proposed scheme leverages phase adjustment and relaying power control to completely eliminate the covert signal at the warden, Willie, thereby enhancing the covertness of the transmission. Furthermore, we derive the optimal power allocation strategy at Alice to maximize the covert data rate while satisfying both the covertness constraint and the QoS requirement at Carol. The numerical results demonstrate that the proposed scheme outperforms existing approaches in terms of the achievable covert data rate, confirming its effectiveness and potential in practical covert communication scenarios.

## Data Availability

The original contributions presented in this study are included in the article. Further inquiries can be directed to the corresponding author.
